# An analysis and validation pipeline for large-scale RNAi-based screens

**DOI:** 10.1038/srep01076

**Published:** 2013-01-16

**Authors:** Michael Plank, Guang Hu, A. Sofia Silva, Shona H. Wood, Emily E. Hesketh, Georges Janssens, André Macedo, João Pedro de Magalhães, George M. Church

**Affiliations:** 1 Integrative Genomics of Ageing Group, Institute of Integrative Biology, University of Liverpool, Liverpool, UK; 2 Laboratory of Molecular Carcinogenesis, National Institute of Environmental Health Sciences, RTP, NC 27709, USA; 3 Department of Genetics, Harvard Medical School, Boston, MA 02115, USA; 4 Current address: Centro de Investigação em Ciências da Saúde, Faculdade de Ciências da Saúde, Universidade da Beira Interior, Covilhã, Portugal.; 5 Current address: Department of Biochemistry, Groningen Biomolecular Sciences and Biotechnology Institute, Department of Neuroscience, European Research Institute on the Biology of Ageing, University Medical Centre Groningen, University of Groningen, Nijenborgh 4, 9747 AG, Groningen, The Netherlands.; 6 These authors contributed equally to this work.

## Abstract

Large-scale RNAi-based screens are a major technology, but require adequate prioritization and validation of candidate genes from the primary screen. In this work, we performed a large-scale pooled shRNA screen in mouse embryonic stem cells (ESCs) to discover genes associated with oxidative stress resistance and found several candidates. We then developed a bioinformatics pipeline to prioritize these candidates incorporating effect sizes, functional enrichment analysis, interaction networks and gene expression information. To validate candidates, we mixed normal cells with cells expressing the shRNA coupled to a fluorescent protein, which allows control cells to be used as an internal standard, and thus we could detect shRNAs with subtle effects. Although we did not identify genes associated with oxidative stress resistance, as a proof-of-concept of our pipeline we demonstrate a detrimental role of Edd1 silencing in ESC growth. Our methods may be useful for candidate gene prioritization of large-scale RNAi-based screens.

Stem cell self-renewal is the process by which stem cells divide to create undifferentiated stem cells to maintain their numbers, generate differentiated progeny and create a stem cell pool which can be used throughout the organism's lifetime^1^,^2^. Stem cells play an important role in response to injury, acting as a repair system, and in the maintenance/turnover of various tissues, and therefore maintenance of stem cell pools is essential^3^. It has, however, been observed, in several tissue types, that the stem cells' numbers, ability to self-renew, and cellular proliferation decrease with age, possibly resulting in reduced function and tissue regenerative capacity^1^ and maybe even contributing to the aging process^4^,^5^. It is thought that various factors contribute to this age-associated cell loss, such as oxidative damage and loss of genomic integrity^6^,^7^,^8^. Therefore, understanding stem cell self-renewal may have implications for aging, regenerative medicine and stem cell treatments.

Embryonic stem cells (ESCs), characterized by their ability to proliferate indefinitely *in vitro* (self-renewal) and differentiate into cells of all three germ layers (pluripotency), are derived from the inner cell mass of the bastocyst^9^,^10^. An equilibrium between survival, self-renewal and differentiation signals is essential for the growth of ESCs^11^. Several signal transduction pathways have demonstrated an important role in ESC self-renewal, for example the leukemia inhibitor factor (LIF), bone morphogenetic protein (BMP), mitogen-activated protein kinase (MAPK) and Wnt pathways^12^,^13^,^14^. Additionally, pluripotency-associated transcription factors aid the control of self-renewal; at the core of the self-renewal transcription network are the homeodomain proteins Nanog, Oct4 and the SRY-related HMG box containing protein Sox2^12^,^13^.

Long-lived mutant worms often exhibit increased resistance to oxidative stress. This led to the hypothesis that stress resistance is a biomarker of organismal longevity^15^. Cells from long-lived mammalian species are also resistant to some forms of stress, such as oxidative stress induced by hydrogen peroxide^16^. Therefore, screening for genes that enhance oxidative stress resistance may lead to the identification of novel genes related to aging and longevity. This approach has been successfully demonstrated in worms^17^ whereas in mammals such studies are missing.

Large-scale RNAi-based screens are a major technology to study cellular processes, including stem cell biology^12^,^18^,^19^,^20^,^21^. However, such screens have several bottlenecks and difficulties^19^,^21^. Specifically, given their noisy nature, large-scale loss-of-function screens require adequate prioritization of candidate genes from the primary screen. For example, bioinformatics methods such as network-based approaches are an emerging technique to prioritize candidate genes^22^. Appropriate methods for validation of promising candidates is also essential given that many loss-of-function phenotypes can be subtle.

In this work, our aim was to perform a genetic screen for genes associated with oxidative stress resistance. By employing mouse ESC, we also aimed to gain insights into the molecular mechanisms involved in stem cell self-renewal, pluripotency and the signaling pathways responsible for differentiation. Understanding these mechanisms is crucial to develop viable stem cell therapies, as well as giving an insight into development, cancer and aging^1^,^14^. Therefore, we performed an RNAi-based screen in ESCs for oxidative stress resistance using the Hannon-Elledge Library and identified several candidates. We then developed a bioinformatics pipeline to prioritize these candidates that not only takes into account effect sizes but also incorporates functional enrichment analysis, interaction networks and gene expression information. To validate candidates with modest effects on cell growth we employed a flow cytometry-based proliferation assay. Although we failed to validate genes associated with oxidative stress resistance, as proof-of-principle of our pipeline, we demonstrate a detrimental role of Edd1 silencing in ESC growth. Our methods may be useful for candidate gene prioritization of large-scale RNAi-based screens.

## Results

### Initial RNAi-based pooled screen for genes affecting resistance to oxidative stress

An initial screen was performed to identify candidate genes involved in the ability of ESC to survive under oxidative stress. The screen employed the Hannon-Elledge whole-genome shRNA library^23^; more specifically we used a fraction of the library with 6,796 shRNAs. These shRNAs are integrated into the genome, expressed from a promoter, and recognized as miRNAs in the miRNA pathway, resulting in gene silencing of a desired gene^23^. Since the library we used contained more than one shRNA per gene, around 2,000 to 3,000 genes were targeted.

**Figure 1:**
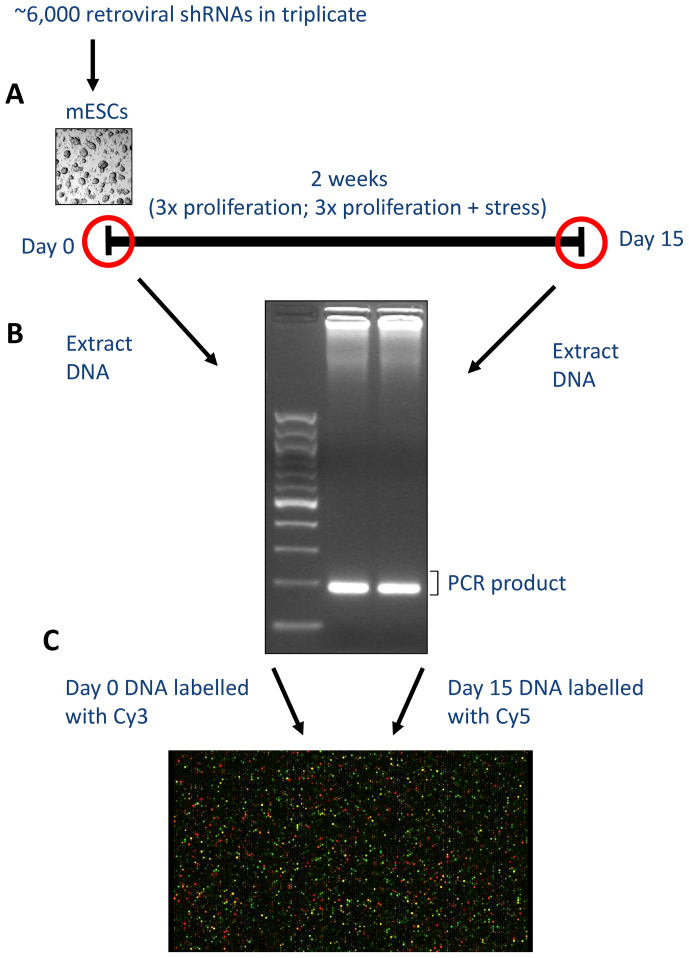
Outline of the pooled screen to find genes associated with susceptibility to oxidative stress. A. Mouse ESCs are transduced with shRNAs and allowed to proliferate for two weeks in triplicate plus allowed to proliferate for two weeks while being exposed to oxidative stress at regular times in triplicate. B. Genomic DNA is extracted from cells at the start and end of the experiment, shRNAs are PCR amplified and gel extracted. C. Samples are labeled with dyes and hybridized to a microarray. shRNAs enriched during the screen give a red spot while those depleted give a green spot.

Mouse ESCs from the CCE line were virally transduced in triplicate by adding a mixture of lentiviruses as vectors for the shRNAs which integrated into the cells' genome, approximately one copy per cell. Following antibiotic selection, cell pellets were frozen to serve as the initial time point. Then, for each replicate, cells were cultured for two weeks with and without regular exposure to oxidative stress (see Materials and Methods). The use of a control where cells proliferate without being exposed to oxidative stress is necessary to eliminate genes selected due to proliferation effects from the screen for oxidative stress resistance. Genomic DNA was extracted from cells at the end of the experiment and at the start (Figure 1). The DNA integrated shRNA encoding sequences which were then PCR amplified and gel extracted. The DNA isolated at the start of the experiment was labeled with the Cy3 dye, and the Cy5 dye was used for the DNA isolated at the end of the experiment. Both were hybridized to a microarray using matching samples from the beginning and end of the experiment.

The green and red signals were quantified from the microarray and ratios ln(red signal/green signal) calculated. As such, the ln(red signal/green signal) ratio of shRNAs knocking-down genes that have a positive effect on cell growth will diminish due to this effect, while shRNAs knocking down genes with a negative effect on cell growth will increase. Similarly, for the experiment focused on oxidative stress, the ln(red signal/green signal) ratio will indicate genes increasing or decreasing susceptibility to oxidative stress. An outline of the experiment is shown in Figure 1.

### Prioritizing genes for experimental validation

A value counting method was used to identify and rank significant genes, as this avoids problems with outliers and minimizes the noise intrinsic to the pooled screen. There will be considerable noise in the experiment, resulting in fluctuations in the results across replicates, and our value counting method for selecting candidates minimizes the impact of such noise by not taking into account the effect sizes. As such, to identify significant genes, for each probe we counted the number of times the ln(red signal/green signal) exceeds a certain positive or negative threshold and calculated the probability that this is a higher number than expected by chance. A false discovery rate (FDR) was estimated by scrambling (see Materials and Methods). Using this approach, the results were not statistically significant for identifying genes affecting susceptibility to oxidative stress (not shown). We therefore decided to focus on testing candidate genes for association with stem cell growth instead of for association with stress response. Our results for stress resistance are given in the Supplementary Dataset 1 if other researchers wish to further explore them.

For identifying candidate shRNAs with effects on cell growth, the microarray results from all six experiments (three replicates where cells proliferate and three where cells proliferate with stress exposures; Figure 1) were combined in order to increase the statistical power. Using the above value counting method, a cutoff of 5 (out of 6) significant replicates above or below the threshold yielded statistically significant results at FDR < 0.05 (Table 1). In total, 23 over- and 60 under-represented genes were identified as significant; 1–2 false positives would be expected at FDR < 0.05 which we think is appropriate.

Another criterion for candidate gene prioritization was the association of a gene to Gene Ontology (GO) terms enriched among top hits from the screen (see Materials and Methods). The GO identifiers and terms at a P-value of 0.005 (FDR = 0.08 and 0.06 for over- and under-represented genes respectively) are shown in the Supplementary Material Table 1. Briefly, for over-represented genes we obtained categories related to phosphate, ATP and phosphorylation and for under-represented the proteasome. To exclude any biases from a pre-selection of genes for inclusion in the shRNA library, we also employed the Database for Annotation, Visualization and Integrated Discovery (DAVID)^24^ using default parameters and the genes on the microarray as background. Searching for enriched pathways below a FDR of 5%, MAPK signaling was found for over-represented genes, the proteasome again for under-represented.

We used STRING to derive a network view of our top cell growth results (see Supplementary Material Figure 1). While many proteins were not or weakly connected, there were two distinct dense parts of the network, one built around Tcf4, Pparg and including edges to Hdac2 and Hdac3 and another around Psma1 and Psma5, strongly linked to Pak1. We assumed that a gene with a high degree of connectivity in the network strengthens evidence for the importance of that gene in mechanisms related to stem cell growth.

To further select candidates to be experimentally tested, we took into account if a gene was also significant at the 6of6 criterion (i.e., significant in 6 of the 6 replicates) or significant at the 5of6 criterion with more than one probe and if it was associated with meaningful GO-categories. As a meaningful GO-category we defined one that describes a distinct cellular process, not a function that can be found in many different pathways. Enriched meaningful functional categories were “cell differentiation”, “apoptosis” and those related to proteasome function (see Supplementary Material Table 1).

For over-represented candidates we selected Rnf31, Pkn2, Map4k5, Csnk1a1 and Ppp3r2 since they all fulfilled the 6of6 criterion, Clk1 because it was found significant by two probes and Map3k1 for its central role in the network (6 connections) and its functional association with “apoptotic mitochondrial changes”. Candidates for which the shRNA was under-represented after 2 weeks we chose Edd1, Hdac3, Phf17, Sqstm1, Mbd2 and Zxda since they all were significant at the 6of6 criterion and were associated with meaningful functional categories. Psma5 was chosen because it was found significant by two probes and for its role in proteasome function and high degree (7 connections) in the network. Interestingly, there was only a modest overlap with top genes from simply ranking genes by average log changes (not shown).

We also checked the expression of the selected candidates in early embryonic stages and stem cell lines in public datasets. If the expression of a gene (more precisely: its percentile rank within the sample) was at a low level for t = 0 in a differentiation time course/for undifferentiated cells and the level at other time points/in the embryoid body were clearly higher this raised doubts about whether the gene is expressed in stem cell lines; if it was at background level for most of the time points/also for the embryoid body we did not directly assume this gene to be not expressed in embryonic stem cells without further hints from other analyses. The results are shown in the Supplementary Material Table 2. For all genes except Ppp3r2 and Zxda there was at least one type of evidence for expression in embryonic stem cells; in other words, results from at least one of the databases consulted suggested that the gene was expressed. Even though the data do not unambiguously prove that Ppp3r2 and Zxda are not expressed in stem cells, we excluded these genes from further validation. The final list of candidate genes is given in the Supplementary Material Table 3.

### Experimental validation of candidate genes by assaying for long-term cell growth effects

Our initial analyses comparing the number of cells plated to the number of cells after 3–5 days of growth were unsuccessful (not shown). Briefly, the growth rates of cells for the shRNA-transduced lines over this period was compared to that of un-transduced cells using 3 replicates for each. These lines included one expressing Firefly (FFL) shRNAs as a negative control and Oct4 and Psma1 shRNAs as positive controls. No significant changes in the proliferation rate between the lines could be detected, and though effects in positive controls were noticeable by visual inspection, often the differences were not statistically significant (not shown).

A limitation of standard cell proliferation assays is that for meaningful results the cells have to be in their exponential proliferation phase when counted and splitting the cells is not possible without considerably increasing variation. If sub-culturing was to be avoided, rapidly growing cells like ESC could not be allowed to proliferate longer than 3 or 4 days, even though a longer proliferation time would lead to more significant results if cells could be kept in exponential growth. Therefore we decided to optimize and employ an assay where shRNA lines are mixed with wild-type (wt) cells as an internal standard and monitor their ratio over a longer time. When having an internal standard, splitting becomes possible since any errors or variations in cell numbers between plates during splitting will affect both cell lines.

We employed a construct containing the shRNA linked to turboRFP. This way un-transduced cells were used as an internal standard as these could be distinguished from cells expressing the shRNA by means of fluorescence. In mixtures of transduced and un-transduced cells the proliferation ratios between them are therefore comparable even if different replicates are not plated at exactly the same density or any factors (e.g. trypsinization) affect proliferation or cell death.

**Figure 2:**
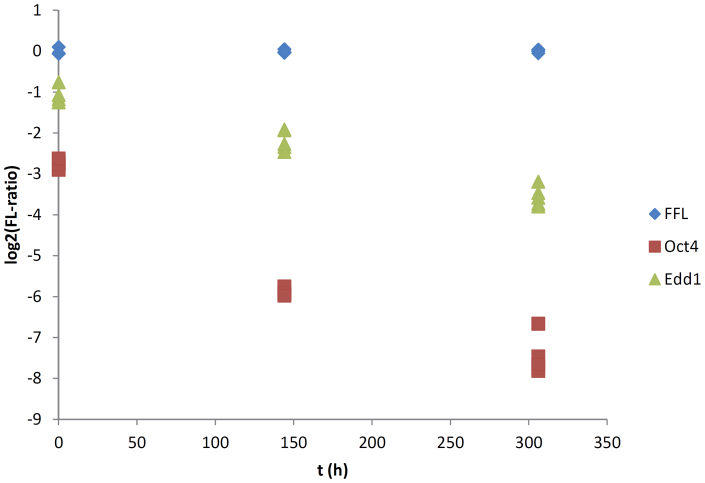
Fluorescence ratio (log2-transformed) of Edd1 (green), Oct4 (red) and FFL (blue) cell lines over time (in hours); each symbol represents a replicate.

Some shRNAs were not successfully cloned or were not available from the Hannon-Elledge library and thus were excluded. For the five remaining candidate genes (Map3k1, Pkn2, Edd1, Map4k5, and Hdac3) and a positive control (Oct4), we mixed equal numbers of cells expressing the shRNA with un-transduced cells and allowed cells to proliferate for two weeks, taking regular measurements *via* flow cytometry to estimate the ratio of cells expressing or not expressing RFP. Cells transduced with FFL were used as negative control. Apart from Oct4, our results showed a much stronger decrease of fluorescent cells in the cell line transduced with the Edd1 shRNA than in all other cell lines (Figure 2). After one week of proliferation there is a 54% ± 17% SD decrease in fluorescent cells while after two weeks a decrease in 81% ± 17% SD was observed.

### Further validation and silencing of Edd1 determined by qPCR

**Figure 3:**
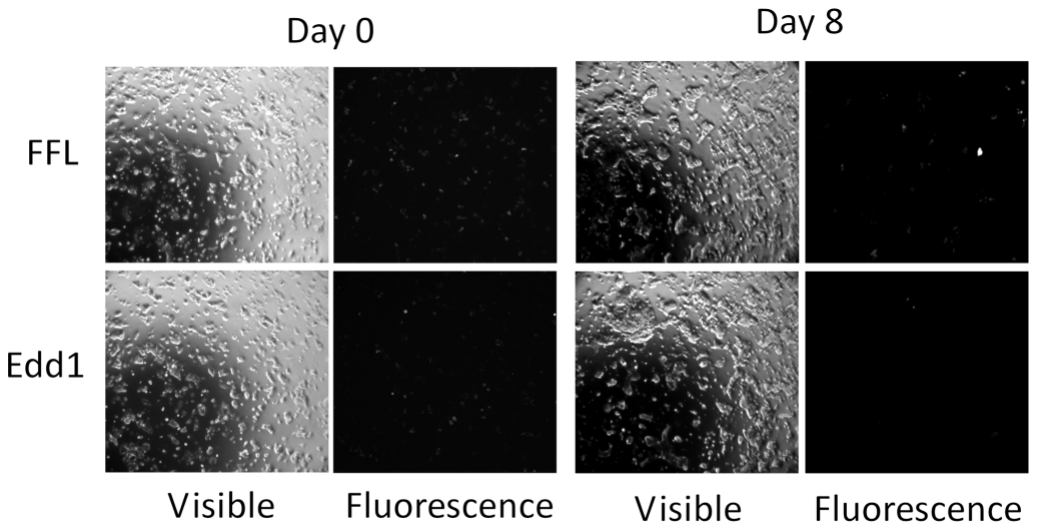
ESC expressing FFL and Edd1 shRNAs (together with RFP) in bright field and fluorescence microscopy. At day 8 the decrease in fluorescence in the Edd1 cells but not in the FFL cells is obvious.

The finding that Edd1 silencing affected cell growth was then repeated in triplicate by following the fluorescence loss of cells expressing the Edd1 shRNA compared to the FFL line using fluorescence microscopy. Clearly, fluorescence-positive cells become depleted after only one week of proliferation (Figure 3). This result was highly reproducible and Su et al., (2011) recently reported similar results^25^. Taken together, these results provide proof-of-principle that our pipeline can detect biologically-relevant results.

qPCR was then used to determine that Edd1 was indeed being silenced in cells expressing the Edd1 shRNA. Robust silencing (nearly 10-fold) of Edd1 was observed, though it should be noticed that a modest, but significant, silencing of Edd1 was also observed in FFL cells (see Supplementary Material Figure 2).

## Discussion

RNAi-based screens in mammalian cells are an increasingly popular tool for the identification of new genes involved in a number of processes. Our experimental design entailed a drug selection step to minimize noise from un-transduced cells, yet this means that shRNAs with dramatic effects on cell proliferation may be depleted by the time the experiments starts and will be missed. Initially, our aim in this work was to identify genes associated with oxidative stress resistance, with the ultimate aim of obtaining stress-resistant mouse ESCs from which to make mice resistant to oxidative stress. Unfortunately, the results for oxidative stress when eliminating cell proliferation effects were not statistically significant, suggesting that more replicates or a larger experimental scale are necessary for this type of approach. It is possible that changes on a system level might be caused by relatively small changes in individual genes. Another hypothesis is that our initial library targeting genes relevant to cancer research may be a contributing factor to our lack of hits related to oxidative stress; a library focused on stress responses and metabolic processes might have been more adequate.

Although our initial goal of identifying genes that affect susceptibility to oxidative stress was not achieved, by treating all six microarrays as replicates we found several candidate genes affecting cell growth. From our shRNA library pooled screen we identified 23 over-represented and 60 under-represented shRNAs significantly (FDRs < 0.05) altered in their abundance during cell proliferation and whose respective target genes are candidates for cell growth effects, respectively, by hindering and promoting cell growth. An advantage of using a value counting method for selecting candidate genes for validation is the insensitivity of this test to outliers. There will be considerable noise in the experiment, resulting in fluctuations in the results across replicates, and our value counting method for selecting candidates minimizes the impact of such noise by not taking into account the effect sizes. We also employed GO categories and network analyses to further prioritize candidate genes and tested if genes were expressed at embryonic stages or in stem cells to further refine our list of candidates.

By their association to (enriched) functional categories, the number of probes by which they were found and their degree in the network of all genes targeted by these 83 shRNAs, we selected 10 candidates for which to validate their role in ESC growth. To assay for modest proliferation effects, we employed a method using flow cytometry to validate our shRNAs, similar to a multi-color competition-based assay previously reported^26^,^27^. The advantage of this flow cytometry method over standard cell counting experiments is that it combines control and experimental lines, which are under exactly the same culture conditions and can be trypsinized. This in turn allows cells to proliferate for a longer time, resulting in a better signal-to-noise ratio. One potential caveat, however, is that transduced cells might affect un-transduced cells secreting factors or other cell-cell interactions.

Using the above method, we observed a marked decrease in fluorescence in our positive control (Oct4) and in Edd1 cells. Edd1 silencing effects on cell growth were highly reproducible and cells with Edd1 silenced clearly became depleted with continuing passaging. The ortholog of the *Drosophila* hyperplastic disc gene (hyd), crucial for cell proliferation during development in flies, Edd1 has been found overexpressed in several cancers and is involved in regulation of DNA damage responses, possibly *via* Chk2^28^. Studies in other cell types have shown that Edd1 regulates DNA damage checkpoints and its disruption can affect cell proliferation and cell cycle, often increasing the percentage of mitotic cells but also inducing cell death^28^,^29^. Edd1-deficient mouse embryos exhibited delayed growth accompanied by a decrease in cell proliferation^30^, in line with our results. More recently, a genetic screen in mouse ESC showed that Edd1 deficiency resulted in growth defects^25^. Therefore, while our results are mostly confirmatory, they provide proof-of-principle that our pipeline can generate phenotypically-relevant results.

In conclusion, we performed an RNAi-based screen for oxidative stress resistance that, although failing to identify genes associated with resistance to oxidative stress, revealed candidates for effects on cell growth which we prioritized with functional, integrative analyses. We developed a flow cytometry method for testing candidates with high sensitivity from which we identified Edd1 as being crucial for cell growth of ESCs. Our methods could be useful for further studies and the role of Edd1 in ESC self-renewal warrants further investigation.

## Methods

### shRNAs and vectors

A subset of the Hannon-Elledge library^23^ with 6,796 shRNAs was employed. The genes targeted by this so called “focus library” were chosen with a focus on cancer research (i.e. targeting genes involved in signaling, cell cycle, etc., where a phenotype was more likely from their knock-down), as described^18^. As a negative control FFL (firefly luciferase) was used since the shRNA targeting FFL does not have a target in murine cells. As positive controls known to affect ESC self-renewal, shRNAs targeting Oct4 and Psma1 were used. The mixture of plasmids containing these different shRNAs was obtained from S. Elledge.

The shRNAs used in this study are second generation, shRNA-mir, designed to silence the specific candidate genes. These are originally contained in the Hannon-Elledge library within a pSM2 vector^20^. They were transferred into pHAGE-Mir, a lentiviral vector designed for efficient gene silencing in ES cells; see Supplementary Material Figure 3 for a map of the vector. The pHAGE-Mir vector uses the pHAGE lentiviral backbone^31^ and expresses a fluorescence marker turboRFP and the shRNA in the same transcript. The RFP expression allows easy monitoring of the amount of transduced cells by FACS analysis. Detailed structure and sequence of the pHAGE-Mir vector will be described elsewhere. pHAGE also contains genes for ampicillin and puromycin resistance for selection in bacteria and eukaryotic cells, respectively.

The inserts of cloned plasmids selected for validation were Sanger sequenced by the University of Sheffield Core Genomics Facility sequencing service. The primer sequence used was 5'-CACGAGATGGCTGTGGCCAAG-3'. The resulting sequence was compared to the expected sequence as provided by the Elledge group.

### Transfection of packaging cell line

The 293T packaging cell line^32^ was transfected with vectors encoding virus particles and pHAGE-shRNA by lipofection with the TransIT-293 Transfection Reagent (Mirus) according to manufacturer's instructions. We transfected plasmids at ratios of pHAGE-shRNA : PM2 : Rev : Tat : VSVG = 10 : 1 : 1 : 1 : 2, where PM2, Rev, Tat and VSVG stand for expression plasmids coding for viral Gag-Pol, Rev, Tat and G-protein of the vesicular stomatitis virus (VSVG). pMD2.G and psPAX2 (Addgene plasmids 12259 and 12260, respectively) were used as packaging plasmids. Medium was changed the next day to DMEM-F12 (Gibco) with 10% FBS, penicillin and streptomycin. One day later if cells appeared to be red due to the expression of turboRFP and (nearly) confluent the supernatant was collected and used for transfection of ESCs. The supernatant contained replication-incompetent lentivirus, as described^31^.

### Viral infection of embryonic stem cells

Polybrene (Millipore) was added to the viral supernatant to a final concentration of 4.5 μg/ml. ESCs were trypsinized and 8 million cells, according to counting with Coulter Counter Z1 (Beckman Coulter), were resuspended in the viral supernatant and transferred to a 100 mm plate; this procedure was done in triplicate (i.e., three independent infections were performed). The multiplicity of infection (MOI) was 0.5–1, which means that at least 4 million cells were initially transduced, and thus on average each shRNA is represented in >550 cells (assuming a Poisson distribution, no shRNA is expected to be represented in fewer than 450 clones). The plate was centrifuged at 2000 rpm at 25°C for 50 min and cells were incubated at 37°C overnight. The medium was changed to ES-DMEM the next day and to ES-DMEM with 2 μg/ml puromycin the day after. Cells were then cultured as described below, keeping them on ES-DMEM with 2 μg/ml puromycin for about 3 days until sufficient fluorescence intensities were reached and uninfected control plates exhibited widespread mortality. Cells were allowed to recover from the stress induced by puromycin selection for 2 days before proceeding with the experiments.

### ESC culture

Feeder-independent mouse ESCs of the CCE line^33^ at around 50–70 passages were cultured on gelatin-coated plates in ESC-qualified Dulbecco's modified Eagle's medium (ES-DMEM) in a 37°C and 5% CO_2_ incubator, as described before^12^. ES-DMEM was made up from KO-DMEM (Invitrogen), 15% FBS (HyClone), 2 mM GlutaMAX (Invitrogen), 1 mM non-essential amino acids (Invitrogen), 50 U/ml Pen + 50 μg/ml Strep (Invitrogen), 100 μM β-mercaptoetanol (Invitrogen) and 1000 U/ml of LIF (Millipore). Cells were split at about 80% confluence about every other day and medium changed every day in between. Cells were regularly checked for signs of differentiation or infection under an inverted light microscope.

### Design of pooled screen

Day 0 was defined as 6 days after transduction, when 8.5 million cells were employed from each replicate, and cells were then allowed to proliferate for two weeks. In the experiment for oxidative stress resistance, the same procedure was performed, but during these two weeks, the cells were exposed every other day to hydrogen peroxide (Sigma) at 1 mM for 2 hours, as previously described^34^. Hydrogen peroxide was chosen because of its widespread use as a source of oxidative stress; in fact, ES cells have been shown to be sensitive to oxidative stress with hydrogen peroxide^35^. Oxidative stress with hydrogen peroxide was observed to reduce cell numbers by 30% (+/− 7% SD) at 1 mM, 65% (+/− 4% SD) with 1.5 mM and 91% (+/− 2% SD) with 2 mM; also see Supplementary Material Figure 4. An oxidative stress with 1 mM hydrogen peroxide is therefore adequate because it results in a moderate cell death; lower cell death would make it harder to detect resistant clones while higher cell death would decrease the representation of each shRNA and increase the noise in the experiment. Cells were grown in 100 mm plates. Both cell proliferation and oxidative stress resistance experiments were done in triplicate. Figure 1 provides an overview of the pooled screen.

### Microarray to quantify shRNAs

Genomic DNA was extracted from cells at the start and end of the pooled screen experiment and PCRs performed using primers binding to the flanking regions of the shRNA; the primer sequences were TAGTGAAGCCACAGATGTA and TAATACGACTCACTATAGGGAGTGATTTAATTTATACCATT. For each replicate, 80 μg of DNA were used by performing multiple PCRs in parallel and later pooling the PCR products. Takara Hot-Start Taq DNA Polymerase (Fisher Scientific) in a 100 μl reaction volume was used with: <10 μg DNA, 300 nM final concentration of each primer, DMSO 4% and Taq PCR buffer and dNTP mixture at concentrations recommended by the manufacturer. The amplification was performed as follows: 4 minutes at 95°C, followed by 36 cycles of 35 seconds at 94°C, 52 seconds at 52°C and 35 seconds at 72°C, followed by 10 minutes at 72°C. This amplified the different shRNA encoding sequences in proportion to the amount this sequence was present in the cell population. Cy3 and Cy5 were then incorporated to, respectively, DNA from cells at the start and end of the experiment, hybridized to a custom-made microarray (Agilent), containing two probes per shRNA in the library, and scanned using an Agilent microarray scanner, according to the manufacturer's instructions and as described^18^. Supplementary Material Figures 5 and 6 show QC plots from the microarray data.

### Processing of microarray data

Data from the two-color microarray was normalized using Agilent G2567AA Feature Extraction software 9.1, following the manufacturer's instructions. Probes for which the signal of the green channel was < 200 in at least 3 of 6 microarrays were removed to eliminate low confidence probes. The maximum value for the green signal was around 295,000, the median around 1,300. After this selection, 8,845 of the original 12,288 probes were left.

The gene annotation and mappings were downloaded from Codex (http://cancan.cshl.edu/cgi-bin/Codex/Codex.cgi). Probes for which annotation could not be found (24 in total) were discarded from the analysis. Probes matching more than one shRNA sequence were removed. The number of probes excluded during this procedure was 214.

### Statistical analyses

Since there were two probes per shRNA on the microarray, the two (if both passed the intensity threshold) were collapsed by calculating the mean for each replicate. Mean value and standard deviation (STDEV) for the ln(red signal/green signal) of each experiment over all probes were calculated. (Means were −0.09 to −0.04, standard deviations 0.98 to 1.16.) An shRNA was termed over-represented if the ln(red signal/green signal) was above a certain threshold for a certain number of replicates and under-represented if this number of replicates was below a certain threshold. As threshold for each replicate mean + STDEV over all probes and mean – STDEV respectively were chosen. Those probes for which (at least) 4, 5 or 6 of 6 (termed 4of6, 5of6 and 6of6 criterion) values for ln(red signal/green signal) were above/below the mentioned thresholds were selected. (Microarrays from samples subjected to stress and controls were treated as replicates for this purpose to increase sample size). The occurrences of the number of different probes for shRNAs targeting the same gene were also counted.

For all probes, 13% were above mean + STDEV, 14% were below mean - STDEV. By chance the probability *P* of finding a probe at least 4, 5 or 6 times respectively above/below mean +/− STDEV (called “4of6”, “5of6” and “6of6” criterion) was calculated using the cumulative binomial distribution:
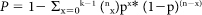


With p = average probability over all probes to be above/below mean +/− STDEV; k = 4, 5 or 6 respectively; n = 6.

By multiplying the probability of finding a probe at the given criterion by the total number of probes one can estimate how many probes are expected to be found by chance. Dividing the number of the found probes by those expected gives the false discovery rate (FDR) which is shown on Table 1. The number of over- or under-represented shRNA candidates closely resembles the number of candidate target genes, since only very few genes (7 for the 4of6 overrepresented, 8 for 4of6 underrepresented, 1 for 5of6 over- and under-represented each and 0 for the others) met the criteria with more than one shRNA.

### Functional enrichment analysis

Functional analysis was done by searching for GO terms that were significantly more associated with over-/under-represented genes than expected by chance. To add GO categories to the corresponding gene a list mapping GO identifiers to all genes was downloaded from NCBI (ftp://ftp.ncbi.nih.gov/gene/DATA/gene2go.gz; 25/08/2009) and all non-mouse genes were discarded. All GO identifiers were added to the list of probes for over- and for under-represented genes. It was counted how many over-represented and how many under-represented genes were found for each GO identifier and how many for the complete list of all genes after collapsing. Only GO identifiers with at least 3 corresponding genes over-/under-represented were used for further analysis.

Significant GO terms were identified using a value counting approach, as previously described^36^. Briefly, the probability *P* that an equal or higher number of over- or under-represented genes is found associated with a given GO identifier more often than expected by chance was calculated using a binomial test:
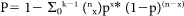


Where k is the number of times a GO identifier was found associated with the over-/under-represented genes, n is the number of times the GO identifier was found associated with all genes and p the probability that GO identifiers were found over-/under-represented. As such, p was calculated by dividing the sum of the number of times all GO identifiers were found associated with over-/under-represented genes by the sum of the number of times they were found associated with all genes.

To assess the significance of the found GO terms and find an appropriate cutoff for *P* considering multiple hypothesis testing we scrambled the ln-ratios of each replicate with respect to each other replicate. The analysis was repeated as with the unscrambled files. Different cutoff values for *P* were tested to find reasonably low FDRs.

As a complement to the above analysis, functional enrichment was studied among over-represented and under-represented candidates from the 4of6 criterion using DAVID^24^. Default options were used and genes represented on the microarray were used as background.

### Gene expression in embryonic stages or stem cells

Initially we tested the expression of candidate genes in the Theiler Stage 4 (TS4) (Blastocyst, Inner cell mass apparent, 2–4 days post coitum (dpc)) and TS5 (Blastocyst (zona-free), 3–5.5 dpc) embryonic stages according to the Mouse Genome Informatics website (http://www.informatics.jax.org/expression.shtml). Afterwards, we checked the number of expressed sequence tags (ESTs) at the Unigene website (http://www.ncbi.nlm.nih.gov/unigene) for the candidates in the blastocyst stage and if not found there in the morula and other embryonic tissues.

We also checked the candidate list for their expression values in the microarray datasets GDS2666 and GDS2667, GDS2668 and GDS2669 as well as GDS2905 and GDS2906 at the Gene Expression Omnibus (GEO). GDS2666 and GDS2667 compare the gene expression in cells of the embryonic stem cell line R1 at different time points towards differentiation to embryoid bodies, GDS2668 and GDS2669 do the same for line J1^37^. GDS2905 and GDS2906 compare gene expression in J1 stem cells and embryoid bodies.

### Network analysis

STRING (http://string-db.org/) is a database of physical and functional protein interactions and can be employed to build a network from a gene list based on this information. We used STRING 8.3 at default settings on a combined list of genes over- or under-represented at the 4of6 criterion.

### Proliferation assay by flow cytometry

To compare growth rates of transduced cells to that of an internal standard of un-transduced cells we mixed them after trypsinization at a ratio of 1:1 for a total of about 700,000 cells. Cell concentrations were determined by counting with a Coulter Counter Z1 (Beckman Coulter) with the lower threshold for particle size set to 0.8 μm.

For flow cytometry, cells were trypsinized and resuspended in about 2 ml of KO-DMEM. To obtain a suspension of single cells samples were pipetted up and down vigorously several times. Flow cytometry was performed on FACSCALIBUR (Becton Dickinson (BD)), controlled by the Cell Quest Pro software, following the manufacturer's instructions. In a first run a side scatter threshold separating presumably intact cells from debris was identified and the same threshold applied in all further runs; 10,000 cells above this threshold were measured per sample. The parameters side scatter (SSC), forward scatter (FSC) and red fluorescence were recorded.

Flow cytometry data were analyzed with WinMDI version 2.9. On a dot plot of SSC vs. FSC the cell population containing presumed living, single cells and excluding dead cells and debris was gated. The same gate was applied for different samples measured on the same day, but the best gate was selected at every day of measurement. For the gated cells on a histogram displaying cell counts vs. fluorescence intensity levels, positive and negative populations were separated at the minimum between both peaks. The intensity value for the border between the peaks was chosen once and kept for all further analyses and always coincided well with the minimum between the peaks. The percentage of fluorescence positive to negative cells was given back by the program. Cells transduced with a shRNA against FFL was used as a negative control while against Oct4 was used a positive control in these experiments.

### qPCR validation

Cells were pelleted and RNA extracted using the RNeasy (Qiagen) standard protocol. The cDNA was generated using the superscript III first-strand synthesis system (Invitrogen) for RT PCR according to the standard protocol (oligo DT). The Edd1 sequence was obtained from Ensembl and the following primers were designed using Perlprimer^38^: Forward: TGCCAAAGCTGAAGTATCTG; Reverse: AATGTCCTGGTTAATGTGCTC. The primers were designed to cross an exon–exon boundary to ensure RNA specificity. ACTB and GAPDH were used as reference genes as they had been employed for this purpose in a previous study in murine ESC (Willems et al., 2006). Standard curves were generated for each assay and indicated that the efficiency of the assay was between 93% and 107% and the R^2^ value was >0.98.

The q-PCR assays were all performed in triplicate using a TaqMan^TM^ ABI PRISM 7500 SDS (Applied Biosystems, Foster City, CA, USA) in 96-well plate format. A 25 μl reaction volume was used per well: 12.5 μl Brilliant II SYBR® Green Low ROX QPCR Master Mix, 10.5 μl cDNA, 1 μl Forward primer (400 nM final conc.), 1 μl Reverse primer (400 nM final conc.). The amplification was performed as follows: 10 minutes at 95°C, followed by 40 cycles of 30 seconds at 95°C and 1 minute at 60°C . The 2^−ΔΔCt^ method^39^ was used to analyse the data, which allows to estimate relative expression normalised by a reference gene.

## Author Contributions

Conceived and designed the experiments: JPM, GMC. Performed the experiments: MP, GH, ASS, SW, EEH, GJ, AM, JPM. Analyzed the data: MP, JPM. Wrote the paper: MP, EEH, GJ, JPM.

## Supplementary Material

Supplementary Information supplementary material

Supplementary Information Supplementary Dataset 1

Supplementary Information Supplementary Dataset 2

## Figures and Tables

**Table 1:** FDRs of candidate shRNAs found over- or under-represented at different criteria. See text for details and Supplementary Material Dataset 2 for full results

	# candidates	FDR
overrep.: 4of6	117	0.158
overrep.: 5of6	23	0.050
overrep.: 6of6	6	0.005
underrep.: 4of6	216	0.100
underrep.: 5of6	60	0.024
underrep.: 6of6	10	0.003
