# Supplementary material for: An analysis and validation pipeline for large-scale RNAi-based screens
Source: Sci Rep. 2013 Jan 16;3:1076. doi: 10.1038/srep01076 (PMC3546318; doi:10.1038/srep01076)

Plank et al. ***An analysis and validation pipeline for large-scale RNAi-based screens***

Supplementary Material:

Table 1: GO-identifiers and terms enriched after two weeks for over-/underrepresentation at FDR< 0.08 and 0.06 respectively.

overrep.		underrep.	
GO:0000287	magnesium ion binding	GO:0000287	magnesium ion binding
GO:0003674	molecular_function	GO:0001843	neural tube closure
GO:0003676	nucleic acid binding	GO:0003676	nucleic acid binding
GO:0003677	DNA binding	GO:0003677	DNA binding
GO:0004672	protein kinase activity	GO:0003700	transcription factor activity
GO:0004674	protein serine/threonine kinase activity	GO:0003713	transcription coactivator activity
GO:0004713	protein tyrosine kinase activity	GO:0004842	ubiquitin-protein ligase activity
GO:0004721	phosphoprotein phosphatase activity	GO:0005515	protein binding
GO:0004725	protein tyrosine phosphatase activity	GO:0005622	intracellular
GO:0005509	calcium ion binding	GO:0005634	nucleus
GO:0005515	protein binding	GO:0005829	cytosol
GO:0005524	ATP binding	GO:0005839	proteasome core complex
GO:0005737	cytoplasm	GO:0006350	transcription
GO:0005739	mitochondrion	GO:0008270	zinc ion binding
GO:0005794	Golgi apparatus	GO:0045449	regulation of transcription
GO:0006468	protein amino acid phosphorylation	GO:0046872	metal ion binding
GO:0006810	transport	GO:0051603	proteolysis involved in cellular protein catabolic process
GO:0006915	apoptosis		
GO:0007165	signal transduction		
GO:0007243	protein kinase cascade		
GO:0007275	multicellular organismal development		
GO:0007399	nervous system development		
GO:0016301	kinase activity		
GO:0016740	transferase activity		
GO:0030145	manganese ion binding		
GO:0030154	cell differentiation		

Figure 1: Network view of top cell growth results. Built using STRING (http://string-db.org/).

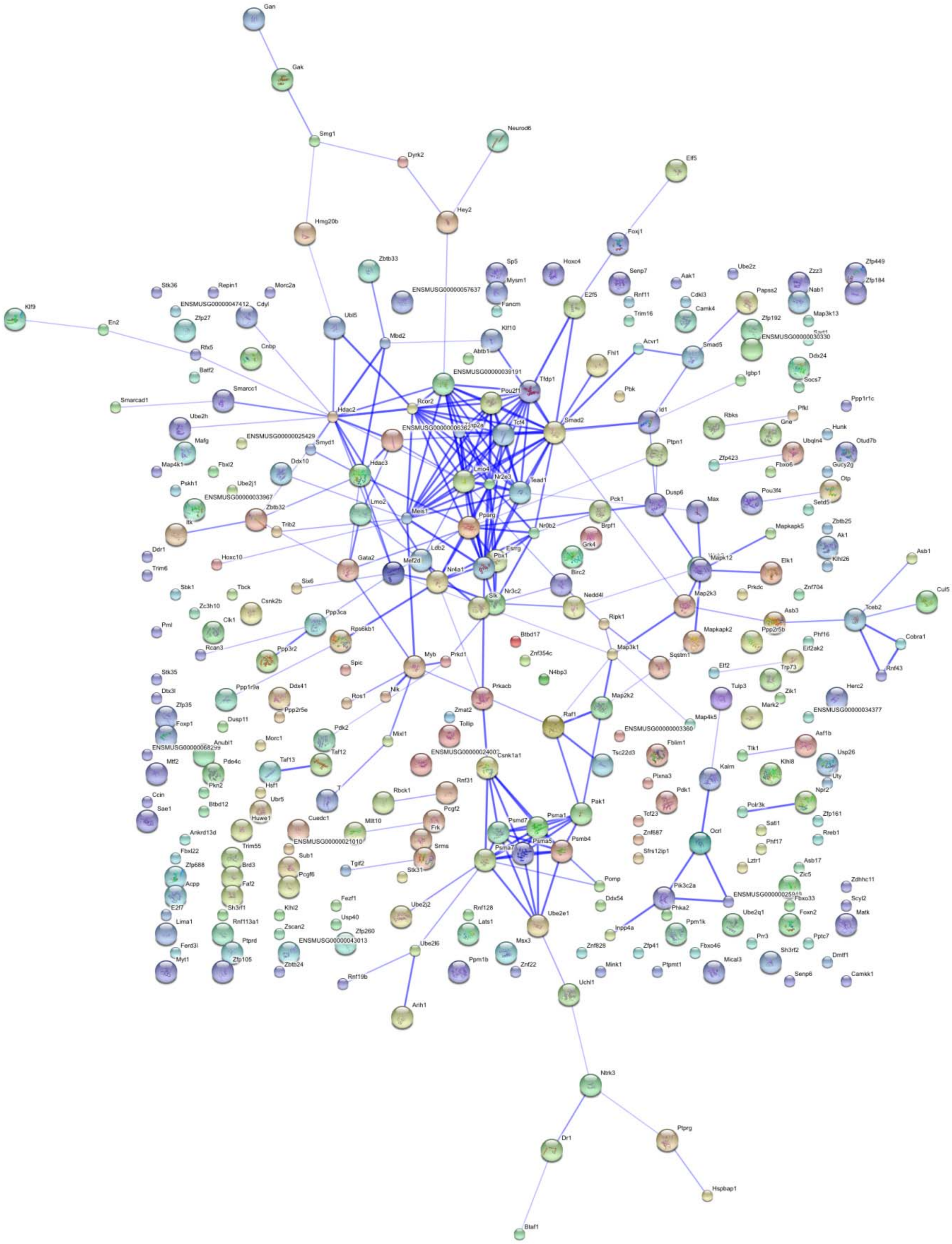

Table 2: Expression levels of selected candidate genes in the blastocyst or if not detected there in the morula and embryonic tissues according to Unigene and percentile position in certain GEO datasets comparing embryonic cells to differentiated cells; "human": information for human homologue; "most low" means that the gene was lowly (<<25%) expressed in both differentiated and stem cells; "undifferentiated low" means the expression of the gene was low in stem cells and clearly higher in differentiated cells; genes with a red background were excluded from the analysis; >x% means all replicates of at least one probe targeting this gene were detected at a higher percentile than x.

	**Unigene: Transcripts per million *in* blastocyst**	**further *Unigene* results**		**GDS2666 and GDS2667(line R1)**	**GDS2668 and GDS2669 (line J1)**	**GDS2905 and GDS2906(line J1)**
Csnk1a1	71			>75%	>75%	>75%
Map4k5	486			>25%	>25%	>25%
Pkn2	100			>75%	>75%	>75%
Ppp3r2	0	0 in embryonic tissue		most low	most low	most low
Rnf31	0	13 in embryonic tissue; 3 in cleavage stage or morula		>75%	>75%	most low
Clk1	28			>75%	>75%	>75%
Sqstm1	271			>75%	>75%	>75%
Psma5	142			>75%	>75%	>75%
Phf17	185			>75%	>75%	>75%
Mbd2	14	human: 0		low	undifferentiated low	undifferentiated low
Edd1	no unigene entry			>75%	>75%	>75%
Hdac3	14			>75%	>75%	>75%
Map3k1	0	13 in embryonic tissue; 0 in cleavage stage or morula		>50%	>25%	>50%
Zxda	no unigene entry			most low	most low	most low

Table 3: Candidate genes for which the shRNAs targeting these genes was significantly over- or underrepresented after two weeks; these genes were chosen for experimental validation.

Candidates for overrepresented genes:

Rnf31	ring figer protein 31
Map3k1	mitogen activated protein kinase kinase kinase 1
Csnk1a1	casein kinase 1, alpha 1
Pkn2	protein kinase N2
Clk1	CDC-like kinase 1
Map4k5	mitogen activated protein kinase kinase kinase kinase 5

Candidates for underrepresented genes:

Edd1	E3 ubiquitin protein ligase, HECT domain containing, 1
Hdac3	histone deacetylase 3
Phf17	PHD finger protein 17
Psma5	proteasome (prosome, macropain) subunit, alpha type 5
Sqstm1	sequestosome 1
Hdac2	histone deacetylase 2
Mbd2	Methyl-CpG binding domain protein 2

Figure 2: Edd1 expression levels compared by qPCR between cells expressing shRNA targeting Edd1, cells expression FFL and control (CCE) cells.

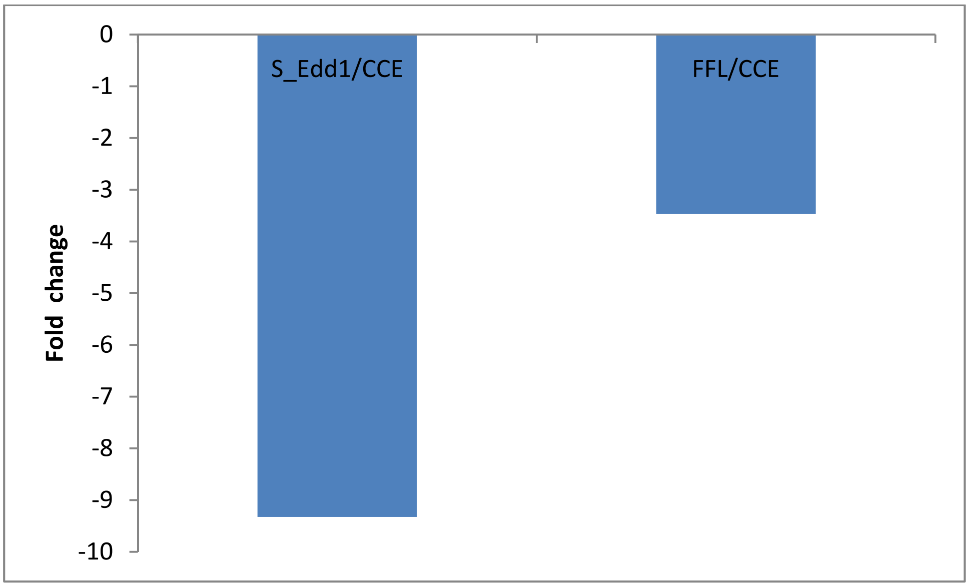

Figure 3: Map of pHAGE-Mir lentiviral vector.

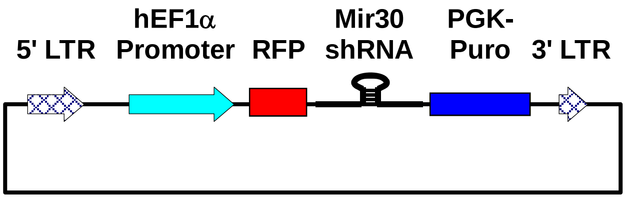

hEF1a promoter: human elongation factor 1a promoter
RFP: red fluorescence protein turboRFP
Mir30-shRNA: shRNA embedded in Mir30 context
PGK-Puro: PGK promoter and Puromycin-resistant gene

Figure 4: ES cells exposed to various levels of oxidative stress: controls, 0.5 mM, 1 mM, 2 mM, 7.5 mM, 10 mM.

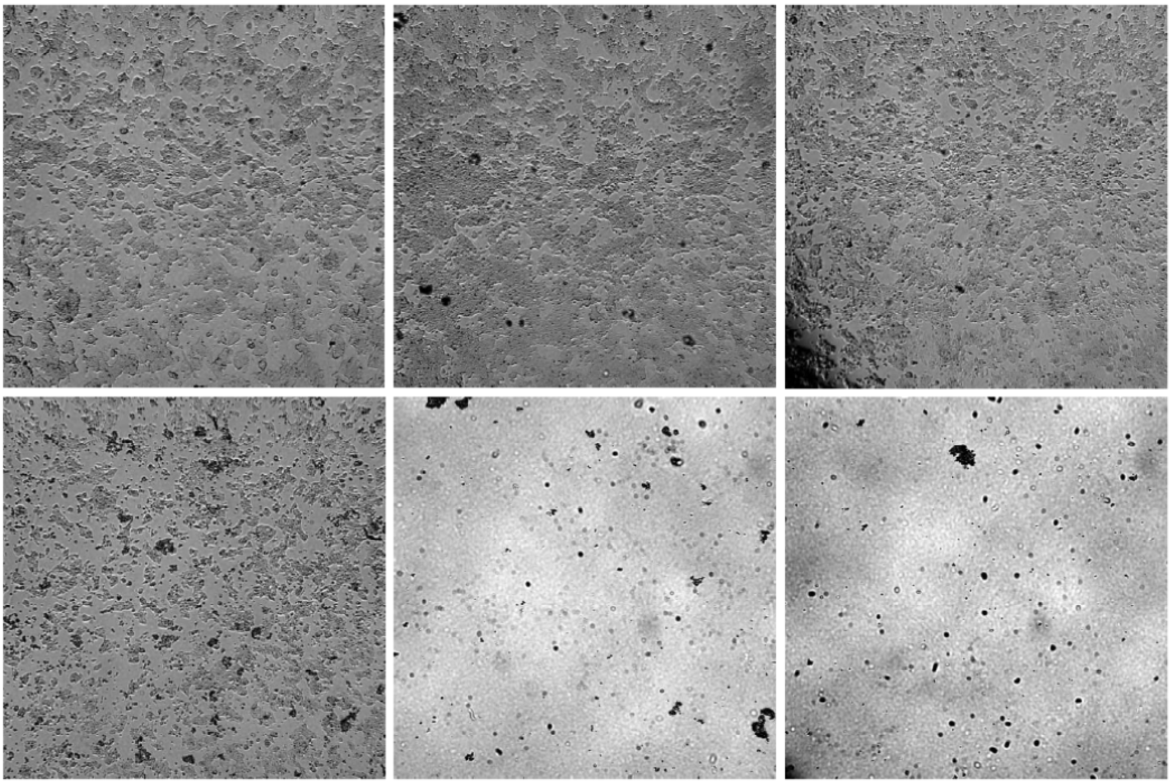

Figure 5: Red and Green Background Corrected Signals (Non-Control Inliers). Proliferation controls are on top and stress on the bottom.

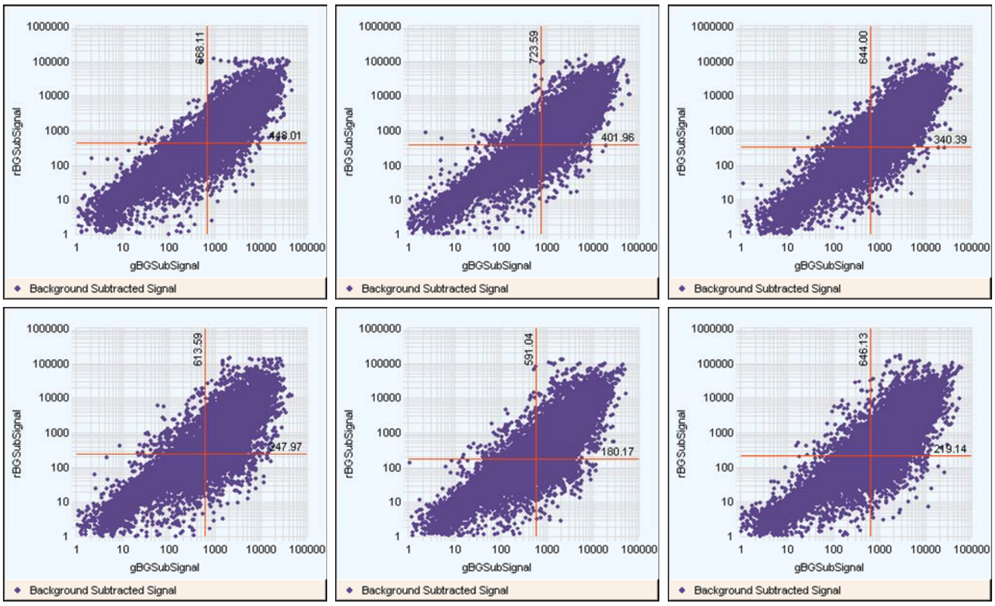

Figure 6: M vs A Plot; LogRatio Versus AvgLogSignal. Proliferation controls are on top and stress on the bottom.

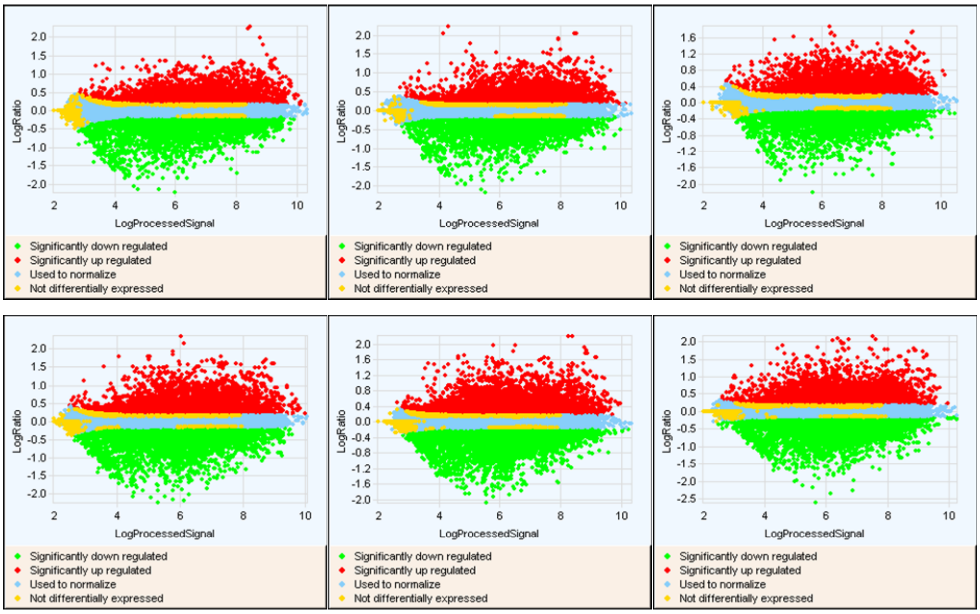

Supplement: Supplementary Information — supplementary material [file srep01076-s1.pdf]
